# Alpha‐mangostin improves endothelial dysfunction in *db/db* mice through inhibition of aSMase/ceramide pathway

**DOI:** 10.1111/jcmm.16456

**Published:** 2021-03-14

**Authors:** Meng Jiang, Shanya Huang, Wang Duan, Qiaoshu Liu, Minxiang Lei

**Affiliations:** ^1^ Xiangya Hospital of Central South University Changsha China

**Keywords:** acid sphingomyelinase, alpha‐mangostin, ceramide, diabetes

## Abstract

Diabetic vascular complications are the leading causes of death and disability in patients with diabetes. Alpha‐mangostin has been reported to have anti‐diabetic capacity in recent years. Here, we investigated the protective function of alpha‐mangostin on endothelium in vitro and in vivo experiments. We also observed that alpha‐mangostin improved impaired endothelium‐dependent vasodilation (EDV) of diabetic animals while it limited the aSMase/ceramide pathway and up‐regulated eNOS/NO pathway in aortas from diabetic mice. Meanwhile, alpha‐mangostin inhibited elevated aSMase/ceramide pathway and reversed impaired EDV induced by high glucose in isolated mouse aortas. In addition, alpha‐mangostin increased phosphorylation of eNOS and NO production in high glucose‐treated aortas. Alpha‐mangostin normalized high glucose‐induced activation of aSMase/ceramide pathway and improved eNOS/NO pathway in endothelial cells with high glucose. In conclusion, alpha‐mangostin regulates eNOS/NO pathway and improves EDV in aortas of diabetic mice through inhibiting aSMase activity and endogenous ceramide accumulation.

## 1 INTRODUCTION

Diabetic vascular complications are characterized by endothelial dysfunction and the leading causes of death and disability in type 2 diabetic patients.^1^ Impairment of NO production is the main contributor to endothelial function disorder in diabetes.^2^ Nitric oxide is produced in vascular endothelium via activation of eNOS. In addition, the eNOS activity might be impaired secondary to ceramide accumulation.^3^, ^4^, ^5^, ^6^

In recent years, our studies have focused on the relationship between ceramide metabolism and endothelial dysfunction in diabetes.^7^, ^8^, ^9^ Our previous data and other’s suggested that inhibiting ceramide accumulation improved the vascular dysfunction in diabetic animals.^4^, ^7^, ^10^ In addition, the improvement of vascular function is mediated by eNOS/NO pathway.^4^, ^5^ Ceramide can be generated by aSMase‐hydrolysed sphingomyelin, which contribute to ceramide accumulation in diabetes.^11^ Activation of aSMase is considered as a pivotal factor in the development of several obesity‐related diseases, including diabetes, atherosclerosis and Alzheimer’s disease.^11^, ^12^, ^13^, ^14^, ^15^ Several lines of evidence indicate that aSMase activity increases significantly in diabetes and inhibiting aSMase/ceramide pathway improves symptoms of diabetes.^11^, ^14^ Therefore, the aSMase/ceramide pathway may be a good therapeutic target for diabetes‐induced vascular dysfunction.

Alpha‐mangostin, a naturally occurring compound isolated from various parts of the mangosteen tree, is able to inhibit the activity of aSMase and has anti‐diabetic capacity.^16^, ^17^, ^18^, ^19^ In addition, alpha‐mangostin is well tolerated at the regular dosage in obese patients from a randomized controlled pilot study.^20^ Furthermore, alpha‐mangostin can improve the body mass index, insulin resistance and oxidative damage of obese people.^20^, ^21^, ^22^ Here, we explored the hypothesis that treatment with alpha‐mangostin‐ameliorated endothelial dysfunction in vivo and in vitro through inhibition of the aSMase/ceramide pathway.

## 2 MATERIALS AND METHODS

### 2.1 Animals study

Ten‐ to 12‐week‐old male wild‐type C57 normal mice and C57BL/KsJ‐diabetic (*db/db*) mice were obtained from Nanjing Biomedical Research Institute. We divided the animals into 3 groups: (i) normal mice control group (NC group); (ii) diabetic mice control group (DC group); and (iii) diabetic mice treated with alpha‐mangostin group (DTM group). DTM group was treated with alpha‐mangostin (10 mg/kg/d, ip), while NC group and DC group just received equivalent solvent. After 12‐week treatment, we measured the glucose, insulin and ceramide levels, aSMase activity, eNOS/NO pathway, EDV in serum and/or vascular tissues. Purified alpha‐mangostin (≥98%) was obtained from Shanghai Aladdin Bio‐Chem Technology Co., LTD. The ethics committee of Central South University approved the present study protocol.

### 2.2 Isolated aortas study

The aortas were isolated from male C57BL/6J mice (10‐12 weeks old) and incubated in DMEM (10% FBS and Penicillin‐Streptomycin). Aortic rings were treated with normal control glucose (NC, 5 mM D‐glucose), high glucose (HG, 30 mM D‐glucose), L‐glucose control (LG, 25 mM L‐glucose + 5 mM D‐glucose), co‐treatment with desipramine (10 μM) and co‐treatment with alpha‐mangostin (15 μM) for 24 hours in an incubator (5% CO_2_; 37°C). After that, we detected the aSMase activity, ceramide content, eNOS expression, NO production and vascular function. Desipramine is able to inhibit aSMase activity and ceramide accumulation.^8^, ^23^, ^24^, ^25^ Here, we used desipramine as an aSMase inhibitor control in this study.

### 2.3 Cell culture and treatment

We isolated the primary aortic endothelial cells from 2 male C57BL/6J mice (6 weeks old).^26^, ^27^ Briefly, the isolated aorta from anaesthetized mouse was perfused with of 1000 U/ml heparin. After connecting tissue was removed, the aorta was placed and digested in collagenase typeⅡ solution at 37°C. After 10 minutes, the endothelial cells were harvested by centrifugation and re‐suspended in endothelial cell growth medium (20% FBS and Penicillin‐Streptomycin) with cell growth kits (Sigma). The primary endothelial cells were identified by positive staining of CD31 (Sigma) and negative staining of α‐SMA (Sigma) (Supplemental Figure S1).

Cells from passages 2 or 3 were seeded in a 6‐well plate and incubated with different treatments. The endothelial cells were divided into 5 groups: (i) normal control glucose (NC, 5 mM D‐glucose) group; (ii) high glucose (HG, 30 mM D‐glucose) group; (iii) HG + 15 µM alpha‐mangostin group; (iv) HG + 10 µM desipramine group; and (v) L‐glucose (25 mM L‐glucose group + 5 mM D‐glucose) group. The alpha‐mangostin concentration was used based on the previous study.^8^ The cultured cells were used for the determination of aSMase activity, ceramide content, NO production and eNOS expression.

### 2.4 siRNA preparation and transfection

For the knockdown experiments, specific siRNA oligonucleotides and control siRNA were purchased from RiboBio. The siRNA molecules were transfected into the cells using transfection kit (RiboBio) following the manufacturer's instructions. The sequences were listed below: aSMase siRNA‐1: 5'‐CCAGUGCAACUACCUACAUdTdT‐3' (sense); aSMase siRNA‐2: 5'‐GCCUCAUCUCUCUCAAUAUdTdT‐3' (sense); and aSMase siRNA‐3: 5'‐GUCUAUUCACCGCCAUCAAdTdT‐3' (sense). We detected the transfection efficiency by flow cytometry (Supplemental Figure S2).

### 2.5 aSMase activity

The aSMase activity was detected by an ultra‐performance liquid chromatography (UPLC) system as previously described.^25^, ^28^ Briefly, we mixed 3 µL of serum, cell lysate or tissue lysate with 3 µL of aSMase buffer. After that, the complex was transferred into the assay buffer and mixed thoroughly. The reaction was stopped by ethanol after samples were incubated for 12 hours. Finally, we tested the product using ultra‐performance liquid chromatography (UPLC) system (Waters).

### 2.6 Ceramide assay

Ceramide content was determined according to a previously described method.^13^, ^25^ First, the lipids were extracted from serum, aorta and cell supernatant. Subsequently, we used 20 µL of 2% Igepal CA‐630 (Sigma) to dissolve the dry lipid. Then, we added 2 µL of ceramide hydrolysis buffer into 2 µL of lipid solution. After 1 hour, we added 56 µL of naphthalene‐2,2‐dicarboxaldehyde buffer into the samples. Then, we incubated the samples at 50°C for 10 minutes. The samples were centrifuged, and the supernatants were measured using the UPLC system.

### 2.7 Vascular function

The vascular function was measured as previously described method.^29^ In brief, aorta was isolated from anesthetized mouse. The aortic segments with a length of 2‐3 mm were incubated in the chamber of Myograph ((Multi Myograph, Danish Myo Technology) with ice‐cold Krebs buffer. The stainless steel wires were guided through the aorta and attached to a force‐displacement device for isometric force determination. The vessels were stretched to tension elicited by 1 g after a 30 minutes equilibration period.

To test the vascular reactivity, aortic rings were exposed to the different treatments. Endothelial relaxation responses were tested with ACh (10^‐8^ M to 10^‐5^ M) using segments previously contracted by phenylephrine (1 μM). Meanwhile, sodium nitroprusside (SNP, 10^‐8^ M to 10^‐4^ M) was applied to test the endothelium‐independent relaxation.

### 2.8 NO assay

NO production was measured using the commercial kit (Jiancheng Bioengineering Institute) as we described.^25^

In situ NO production was detected using DAF‐FM diacetate (Molecular Probes). Isolated aortas were embedded and froze in OCT medium. The cryosections were incubated in PBS with DAF‐FM diacetate (10 μM) for 20 minutes at room temperature. Images were recorded using a confocal microscopy (Leica). Intracellular NO level was assessed using DAF‐FM diacetate (Molecular Probes).^29^ Briefly, the cells with different treatments were rinsed with PBS in the glass chamber. Then, the cells were incubated with DAF‐FM diacetate (10 μM) for 10 minutes at 37°C. Finally, the fluorescence pictures were recorded using a confocal microscopy (Leica). The staining density was quantified using Image‐J software.

### 2.9 ROS assay

Intracellular ROS production was detected using CM‐H2DCFDA (Invitrogen). Briefly, the cells with different treatments were rinsed with PBS in the glass chamber. Then, the cells were incubated with CM‐H2DCFDA (1 μM) for 10 minutes at 37°C. Finally, the fluorescence images were recorded using a confocal microscopy (Leica). The staining density was quantified using Image‐J software.

### 2.10 Glucose and insulin assays

Plasma insulin concentrations were tested with radioimmunoassay kits by radioimmunoassay instrument (USTC). Blood glucose levels were tested using glucose oxidase method.

### 2.11 Western blot analysis

Aortic tissues and endothelial cells were lysed in cold RIPA buffer. After protein quantification, the protein samples (30 μg) were loaded and separated on 8% SDS‐polyacrylamide gel. Subsequently, protein was transferred to PVDF membranes (Millipore) and then blocked by 5% non‐fat milk in TBST. After 30 minutes, the blots were incubated with primary antibodies (eNOS and phospho‐eNOS (Ser1177), 1:1000; Cell Signaling Technology). The next day, membranes were washed for 3 times and applied with secondary antibodies for 2 hours. Finally, the protein was detected using chemiluminescent substrate (Thermo). The immunoblot signals were analysed using the image J software.

### 2.12 Statistical analysis

All data were presented as means ± SEM. Results were analysed using one‐way ANOVA (SPSS 14.0 software). *P* < .05 was considered statistically significant.

## 3 RESULT

### 3.1 Alpha‐mangostin limits aSMase/ceramide pathway and improves metabolic disorder in *db/db* mice

From Table 1, we knew that chronic treatment with alpha‐mangostin decreased the serum aSMase activity and ceramide content in diabetic animals. Meanwhile, we found that α‐mangostin treatment improved blood glucose and insulin levels in diabetic mice. While, alpha‐mangostin did not affect the lipid profile in diabetic mice.

**TABLE 1:** The effects of alpha‐mangostin on metabolic indices

Index	NC group	DC group	DTM group
aSMase (nmol/mL/h)	5.27 ± 0.524	9.66 ± 0.871*	5.38 ± 0.474^#^
Ceramide (ng/mL)	6.83 ± 0.511	10.5 ± 0.583*	7.51 ± 0.354^#^
FBS (mmol/L)	8.47 ± 2.12	29.7 ± 2.87*	15.8 ± 2.59* ^,^ ^#^
Insulin (μIU/L)	8.16 ± 2.04	23.86 ± 3.63*	16.65 ± 2.23* ^,^ ^#^
TG (mmol/L)	0.79 ± 0.15	0.94 ± 0.58	0.82 ± 0.14
TC (mmol/L)	1.42 ± 0.32	3.49 ± 1.11*	3.59 ± 0.46*

All indices were determined after alpha‐mangostin treatment for 12 weeks in NC group (*n* = 6‐8), DC group (*n* = 6‐8), and DTM group (n = 6‐8). TG, triglyceride; TC, total cholesterol; values are means ± S.D.

* *P* < .05, contrasted to normal control group.

^#^ *P* < .05, contrasted to diabetic control group.

### 3.2 Alpha‐mangostin inhibits aSMase/ceramide pathway and improves vascular dysfunction in *db/db* mice

**FIGURE 1:**
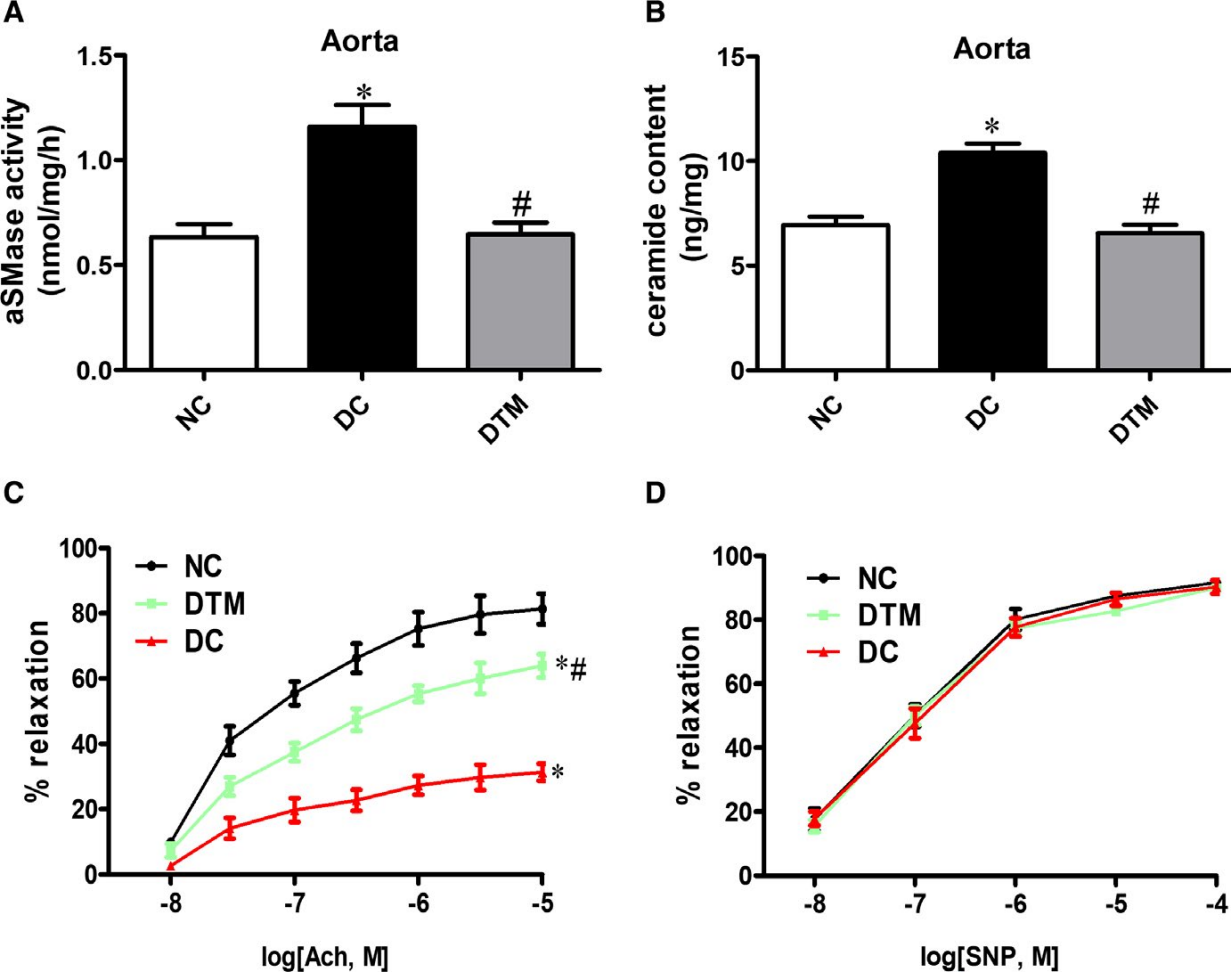
Alpha‐mangostin treatment improves endothelial dysfunction in diabetic mice. A, Aortic aSMase activity of the three groups. B, Aortic ceramide content of the three groups. ACh‐evoked endothelium‐dependent (C) and SNP‐evoked endothelium‐independent (D) vasodilation of aortic segments from diabetic animals treated for 12 weeks with alpha‐mangostin or vehicle. Results are shown as means ± SEM (n = 5‐6). *P < .05 compared to the NC group. #*P* < .05 compared to the DC group

We found that chronic treatment with alpha‐mangostin limited the aSMase activity (Figure 1A) and ceramide accumulation (Figure 1B) in aortas from diabetic animals. Meanwhile, the vascular dysfunction in diabetes was partly restored by alpha‐mangostin treatment (Figure 1C). In addition, the endothelium‐independent vasorelaxation (EIR) was similar among all groups (Figure 1D), which suggested that these changes were the endothelium‐specific defect. Additionally, alpha‐mangostin decreased the aSMase activity and ceramide content in normal mice (Supplemental Figure S3A,B). However, alpha‐mangostin did not change the vascular function in normal mice (Supplemental Figure S3C), which may be due to the fact that the normal mice have intact vascular function.

Therefore, the aSMase/ceramide pathway may mediate the protective effect of alpha‐mangostin on vascular dysfunction in diabetes.

### 3.3 Alpha‐mangostin increases NO generation and eNOS phosphorylation in *db/db* mice

**FIGURE 2:**
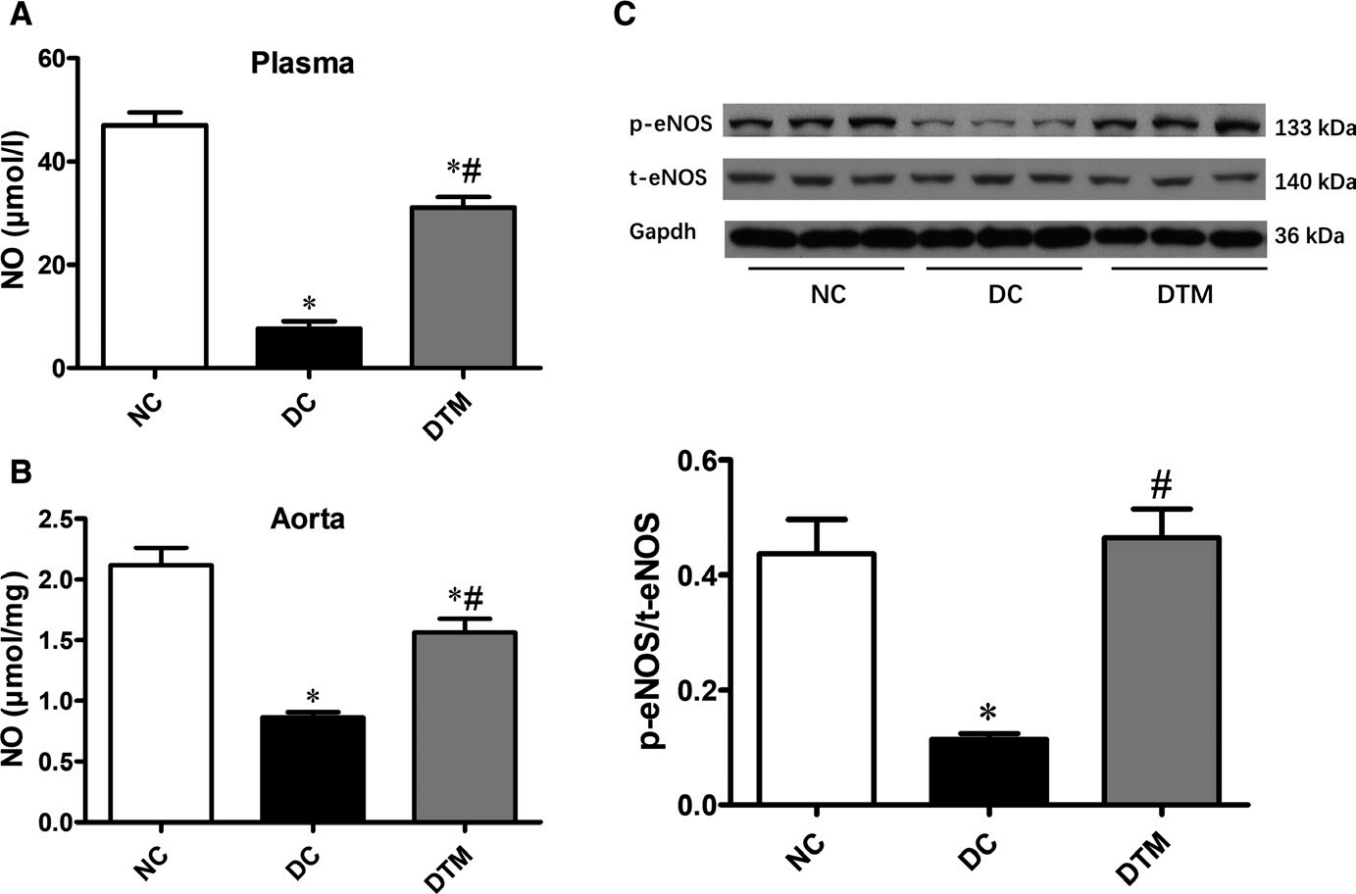
Alpha‐mangostin treatment improves eNOS/NO pathway in diabetic mice. Alpha‐mangostin treatment increased the NO production in the plasma (A) and aortas (B) of *db/db* mice. C, Western immunoblotting of eNOS and phospho‐eNOS in the aortas from different groups. Results are shown as means ± SEM (n = 5‐6). *P < .05 compared to the NC group. ^#^ *P* < 0.05 compared to the DC group

Previous study reported that ceramide accumulation impaired the NO production.^5^ In our study, alpha‐mangostin reserved diminished NO production in diabetic mice (Figure 2A,B). In addition, alpha‐mangostin enhanced the expression of phosphorylated eNOS in diabetic mouse aortas as detected by Western blotting (Figure 2C). Therefore, alpha‐mangostin improves the EVD may through eNOS/NO pathway in diabetic animals.

### 3.4 Alpha‐mangostin limits aSMase/ceramide accumulation and reverses vascular dysfunction in high glucose‐treated isolated aortas

**FIGURE 3:**
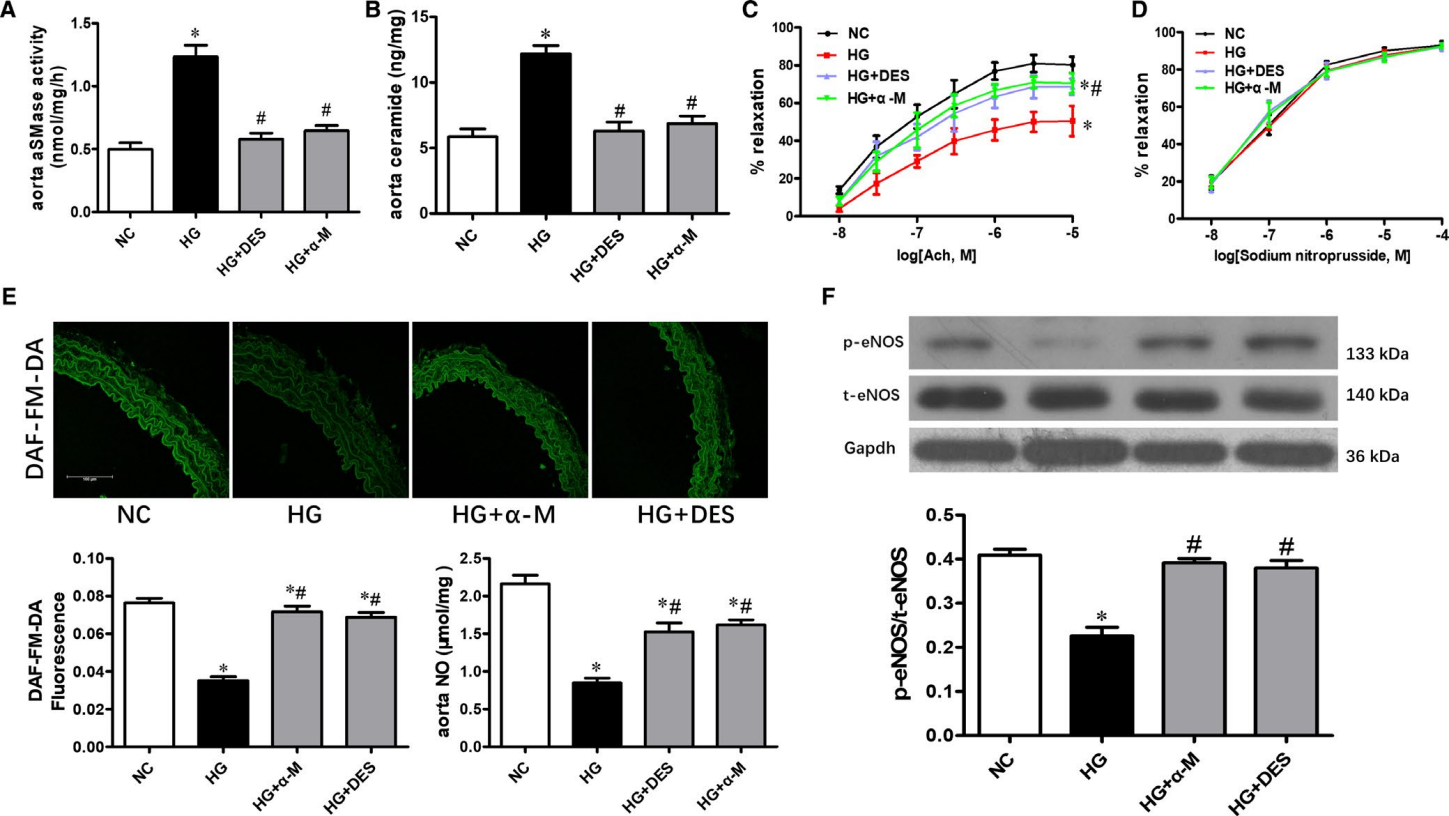
Alpha‐mangostin reverses high glucose‐induced endothelial dysfunction by inhibiting aSMase/ceramide accumulation in isolated aortas. Alpha‐mangostin and desipramine normalized high glucose induced increased aSMase activity (A) and ceramide content (B). High glucose‐mediated impairment of ACh‐evoked vasorelaxation was reversed by alpha‐mangostin or desipramine treatment (C & D). Alpha‐mangostin and desipramine restored the NO production (E) and eNOS phosphorylation (F) in high glucose‐treated mouse aortas. Scale bars represent 50 μm. Results are shown as means ± SEM (n = 5). *P < .05 compared to the NC group. ^#^ *P* < .05 compared to the HG group

Treatment with high glucose for 24 hours raised the aSMase activity and ceramide content, while activation of aSMase/ceramide pathway was prevented by co‐treatment with alpha‐mangostin or desipramine (Figure 3A,B). Alpha‐mangostin or desipramine improved the impaired ACh‐induced relaxations induced by high glucose (Figure 3C). Consistent with in vivo experiment, there was no difference in the SNP‐induced relaxations among these groups (Figure 3D). In addition, high glucose‐mediated impairment of NO production (Figure 3E) and eNOS phosphorylation were restored by alpha‐mangostin or desipramine treatment (Figure 3F).

In conclusion, alpha‐mangostin inhibits aSMase/ceramide pathway and improves endothelial dysfunction in diabetic mice.

### 3.5 Alpha‐mangostin limits aSMase/ceramide accumulation and reverses NO production in endothelial cells with high glucose

**FIGURE 4:**
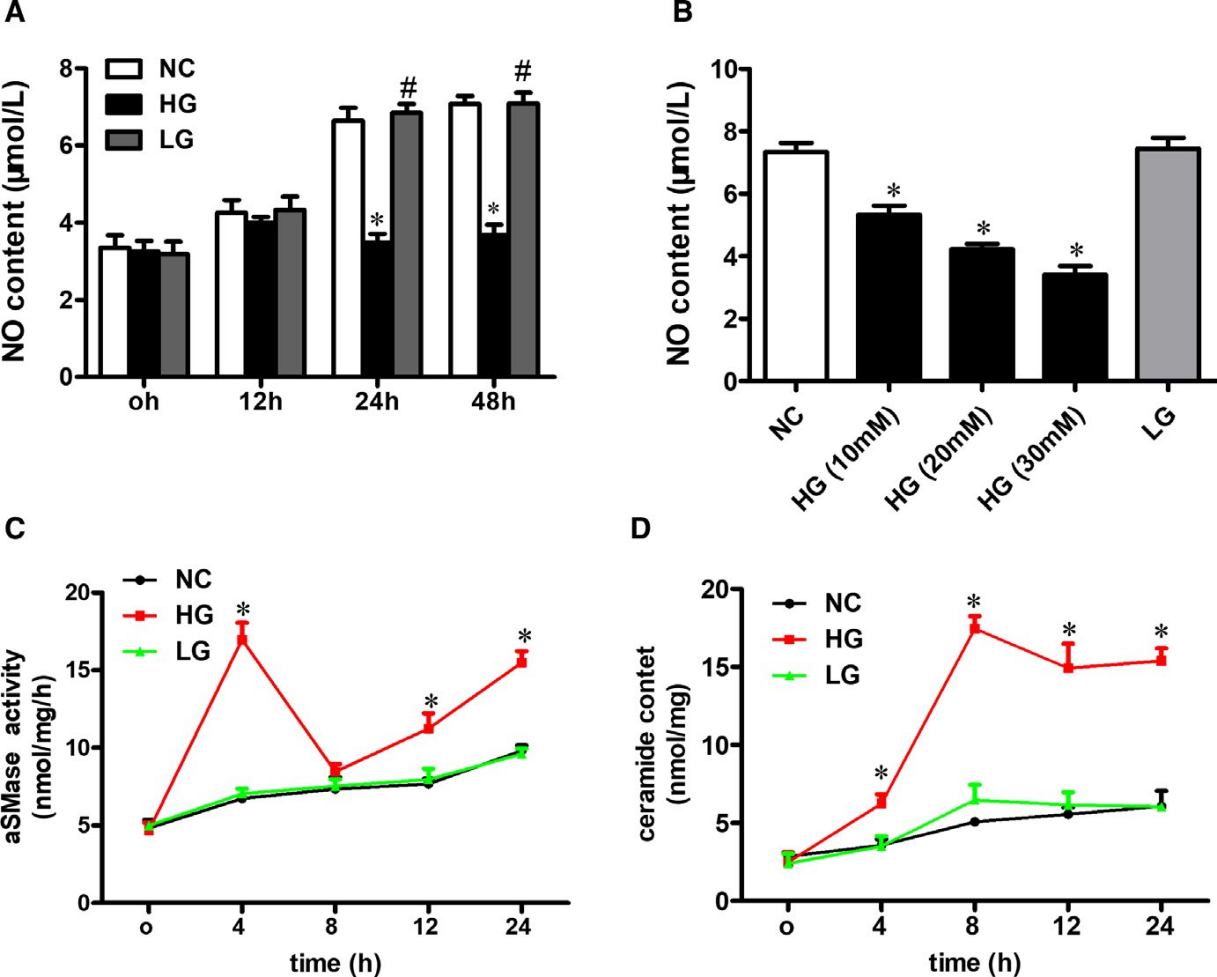
High glucose impairs NO production and interrupts aSMase/ceramide pathway in endothelial cells. A, NO production at different time‐points in cells treated with high glucose. B, NO production at different D‐glucose concentrations in cells. Examination of the effect of high glucose on (C) aSMase activity and (D) ceramide content at different time‐points. Results are shown as means ± SEM (n = 4‐5). *P < .05 compared to the NC group. ^#^ *P* < .05 compared to the HG group

We found that high glucose reduced NO production in a time‐ and dose‐dependent manner in cells (Figure 4A,B). According to the results, we selected a concentration of 25 mM and a time‐point of 24 hours for subsequent experiments. We also observed that high glucose increased the aSMase activity (Figure 4C) and ceramide content (Figure 4D) in endothelial cells. While, L‐glucose did not change the aSMase activity and ceramide levels (Figure 4C,D). These results suggested that the aSMase /ceramide pathway was stimulated within 4 hours treatment and the aSMase/ceramide pathway got stimulated before the NO generation was inhibited.

**FIGURE 5:**
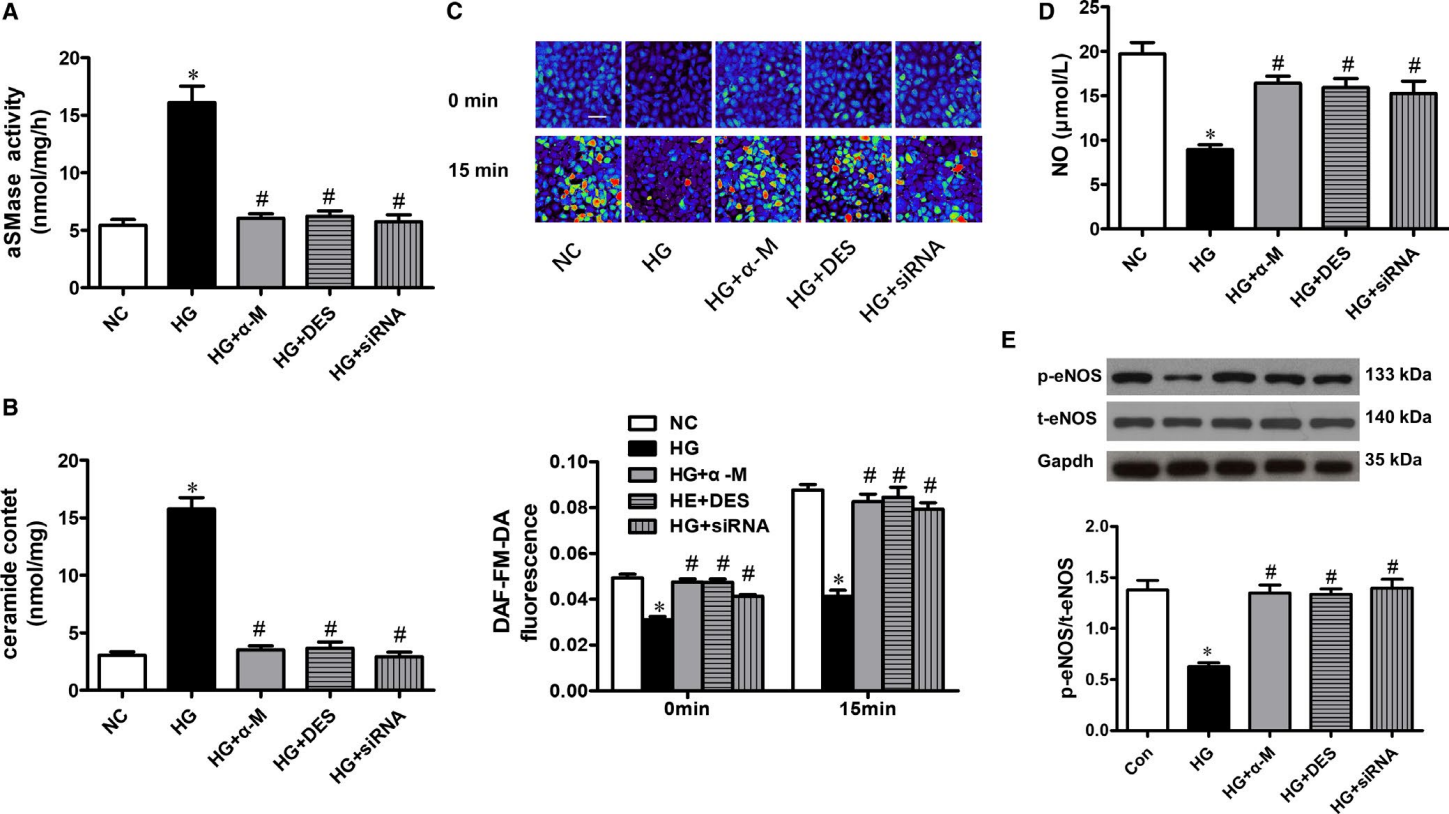
Alpha‐mangostin improves aSMase/ceramide pathway and eNOS/NO pathway in endothelial cells with high glucose. Alpha‐mangostin, desipramine and siRNA restored the elevated aSMase activity (A) and ceramide content (B) induced by high glucose in cell cultures. C, The basal and ACh‐stimulated NO production in the five treatment groups. D, Alpha‐mangostin, desipramine and siRNA reversed the high glucose‐diminished NO production in endothelial cells. E, Alpha‐mangostin, desipramine and siRNA increased eNOS phosphorylation in high glucose‐treated endothelial cells. Scale bars represent 50 μm. Results are shown as means ± SEM (*n* =5). *P < .05 compared to the NC group. #*P* < .05 compared to the HG group

From Supplemental Figure S4, we knew that siRNA decreased the aSMase expression and activity. In addition, we found that the up‐regulation of aSMase/ceramide pathway stimulated by high glucose in endothelial cells was reversed by treatment with alpha‐mangostin, desipramine and siRNA (Figure 5A,B).

High glucose significantly lowered the basal and ACh‐stimulated NO production (Figure 5C,D). The negative effect of high glucose was prevented by treatment with alpha‐mangostin, desipramine and siRNA in endothelial cells.

In addition, the down‐regulation of eNOS phosphorylation induced by high glucose in endothelial cells was inhibited by treatment with alpha‐mangostin, desipramine and siRNA (Figure 5E).

In conclusion, alpha‐mangostin regulates the eNOS/NO pathway through inhibiting aSMase activity and endogenous ceramide accumulation.

## 4 DISCUSSION

The present study shows for the first time that in vivo treatment with alpha‐mangostin improves the endothelial dysfunction and reduces aSMase activity, ceramide content in diabetic animals. We further examined the underlying mechanisms related to aSMase/ceramide pathway and EDV in vivo and in vitro. These results extend our previous studies in which we reported that inhibiting ceramide synthesis improved arterial dysfunction in diabetic rats^7^, ^25^ and inhibiting aSMase activity reversed NO levels in endothelial cells with high glucose.^9^ We now demonstrate that these effects can be recapitulated in isolated vessels, *db/db* mice and cultured cells.

Prior studies revealed that inhibition of ceramide accumulation ameliorated obesity‐related diseases, such as diabetes, hypertension, cardiovascular disease and non‐alcoholic fatty liver disease.^30^, ^31^, ^32^ In recent years, we and other researchers have found that blunting ceramide biosynthesis improved endothelial dysfunction in animal studies.^4^, ^5^, ^7^ Ceramide is a pivot signalling molecular in sphingolipids metabolism and can be generated through three main pathways, including sphingomyelin degradation, de novo synthesis and generation from sphingosine.^33^ Sphingomyelin is the predominant sphingolipid species in cellular membranes. Sphingomyelin hydrolysis is an main source for ceramide generation in diabetes, and aSMase is the key enzyme in this process.^14^, ^34^, ^35^, ^36^ Activation of aSMase is found in cells with high glucose, diabetic patients and diabetic animals.^14^, ^37^, ^38^ In addition, elevated aSMase activity leads to ceramide accumulation and vascular dysfunction in diabetic animals.^11^ Inhibiting aSMase activity by pharmacological or genetic approaches could reserve negative effects of high glucose and/or diabetes.^8^, ^9^, ^14^, ^38^ In summary, aSMase signalling may have an important role in the development vascular dysfunction in diabetes.

Alpha‐mangostin was reported to improve the development of diabetes by inhibiting oxidative stress‐related tissue damage.^16^, ^19^, ^20^, ^39^, ^40^ In addition, alpha‐mangostin was reported as a competitive inhibitor of aSMase^41^ and could inhibit aSMase activity in vivo and in vitro experiments.^8^, ^17^, ^42^, ^43^ Our prior studies indicated that alpha‐mangostin reversed the negative effects of high glucose in cell culture and ameliorated nephropathy in diabetic rats through inhibiting the aSMase activity^8^, ^43^. However, the effect of alpha‐mangostin on vascular dysfunction in diabetes remains undefined.

Here, we observed that alpha‐mangostin improved glucose control and insulin levels in diabetic mice. Amporn et al. reported that alpha‐mangostin was able to improve blood glucose, insulin resistance and diabetic retinopathy in diabetic animals.^16^ Giri et al. found that alpha‐mangostin attenuated hyperglycaemia and diabetic sexual dysfunction in diabetic rodents;^19^ Mikiko et al. showed that mangosteen extract (the main phytochemicals present in mangosteen are alpha‐mangostin) was able to prevent insulin resistance in obese patients.^20^ Welly et al. demonstrated that alpha‐mangostin improved blood glucose and islet β‐cells damage in diabetic mice.^44^ Our previous data suggested that alpha‐mangostin was able to ameliorate blood glucose and diabetic nephropathy.^43^ We knew that alpha‐mangostin was able to inhibit aSMase/ceramide pathway from the present study. Inhibition of ceramide synthesis was able to attenuate insulin resistance from previous studies.^33^, ^45^, ^46^, ^47^ Other researchers also indicated that alpha‐mangostin could improve oxidative stress and islet β‐cell damage.^19^, ^44^ These may explain why alpha‐mangostin can ameliorate insulin and blood glucose levels. However, further molecular mechanisms of the protective role of alpha‐mangostin in diabetes need to be studied.

Here, we mainly focus on detecting the effects of alpha‐mangostin on aSMase/ceramide pathway and vascular function in diabetes. In this study, we found that alpha‐mangostin inhibited the aSMase/ceramide pathway and prevented endothelial dysfunction in diabetic animals. In addition, the improved relaxations were accompanied by increased NO release and restored phosphorylation of eNOS in aortas from diabetic mice, which is consistent with previous investigations.^4^, ^5^ These are advantageous findings, because we show for the first time that alpha‐mangostin ameliorates vascular function in animals with diabetes.

Finding from isolated vessels suggested the impaired EVD induced by high glucose could be prevented by alpha‐mangostin or desipramine treatment, which indicates that up‐regulating aSMase/ceramide pathway can induce vascular dysfunction in a tissue‐autonomous manner. Further support comes from experiments on cultures of cells in which the elevated aSMase/ceramide pathway and reduced eNOS phosphorylation induced by high glucose was reversed by alpha‐mangostin treatment. Meanwhile, gene silencing of aSMase genes or inhibition of aSMase also achieved the similar results.

Alpha‐mangostin could improve oxidative stress in diabetic animals, and NO generation in endothelial cells is much impacted by reactive oxygen species (ROS) production.^48^ In addition, aSMase/ceramide pathway up‐regulation induce ROS generation in a variety of cells, including human aortic smooth muscle cells, endothelial cells and macrophages.^49^ Therefore, we detected whether alpha‐mangostin was able to regulate ROS production in endothelial cells. We found that the high glucose‐induced ROS production was inhibited by treatment with alpha‐mangostin, desipramine and siRNA in endothelial cells (From Supplemental Figure S5). This suggests that alpha‐mangostin and aSMase/ceramide pathway may modulate NO production by regulating ROS production. This is an interesting finding. We will explore the relationship among alpha‐mangostin, aSMase/ceramide pathway, ROS production and eNOS/NO signalling in detail in the future study.

In brief, the accumulation of ceramide decreases phosphorylation of eNOS and NO production, which impairs vascular function in diabetes. Activation of aSMase is an important reason of ceramide overproduction in diabetes. Alpha‐mangostin is able to inhibit the activity of aSMase in diabetic animals, isolated aortas and endothelial cells with high glucose. Therefore, alpha‐mangostin improves vascular dysfunction and suppresses endogenous aSMase/ceramide accumulation in diabetes. In addition, the beneficial effects of alpha‐mangostin were mediated by eNOS/NO pathway.

In conclusion, aSMase/ceramide accrual in diabetic mice impairs eNOS/NO pathway and vascular function in a tissue‐autonomous manner. Inhibition of aSMase reverses vascular dysfunction in diabetic mice, isolated vessels and cultured cells. Our present results provide the first evidence that alpha‐mangostin is effective in ameliorating endothelial dysfunction by inhibiting the aSMase/ceramide accumulation and eNOS/NO pathway in diabetes. Our findings further suggest a therapeutic potential of alpha‐mangostin in alleviating diabetic vascular complications.

Although we have some advantageous findings, there are some limitations in the study. First, we only use male mice in the study. Therefore, we cannot indicate whether the sex difference would induce any possible rift results. Second, the present study does not explore how the aSMase/ceramide pathway affect the eNOS/NO signalling, which deserves future investigation. Last but not least, we just use the db/db mouse as the diabetic animal model with endothelial dysfunction. It will be more comprehensive if we also use another animal model, such as diabetic mouse hindlimb ischaemia model.

## CONFLICT OF INTEREST

The authors declare no conflict of interest.

## AUTHOR CONTRIBUTIONS

**Meng Jiang:** Data curation (equal); Formal analysis (equal); Investigation (lead); Methodology (equal); Software (equal); Validation (equal); Visualization (equal); Writing‐original draft (lead). **Shanya Huang:** Data curation (equal); Investigation (equal); Methodology (equal); Software (equal); Writing‐original draft (equal). **Wang Duan:** Data curation (equal); Investigation (equal); Methodology (equal); Software (equal). **Qiaoshu Liu:** Data curation (equal); Investigation (equal); Methodology (equal); Software (equal). **Minxiang Lei:** Conceptualization (lead); Data curation (lead); Formal analysis (lead); Funding acquisition (lead); Methodology (equal); Project administration (lead); Resources (lead); Supervision (lead); Writing‐review & editing (lead).

## Supporting information

Fig S1

Fig S2

Fig S3

Fig S4

Fig S5

## Data Availability

The data used to support the findings of this study are available from the corresponding author upon request.
