# Supplementary figures and images for: Alpha‐mangostin improves endothelial dysfunction in db/db mice through inhibition of aSMase/ceramide pathway
Source: J Cell Mol Med. 2021 Mar 14;25(7):3601–9. doi: 10.1111/jcmm.16456 (PMC8034454; doi:10.1111/jcmm.16456)

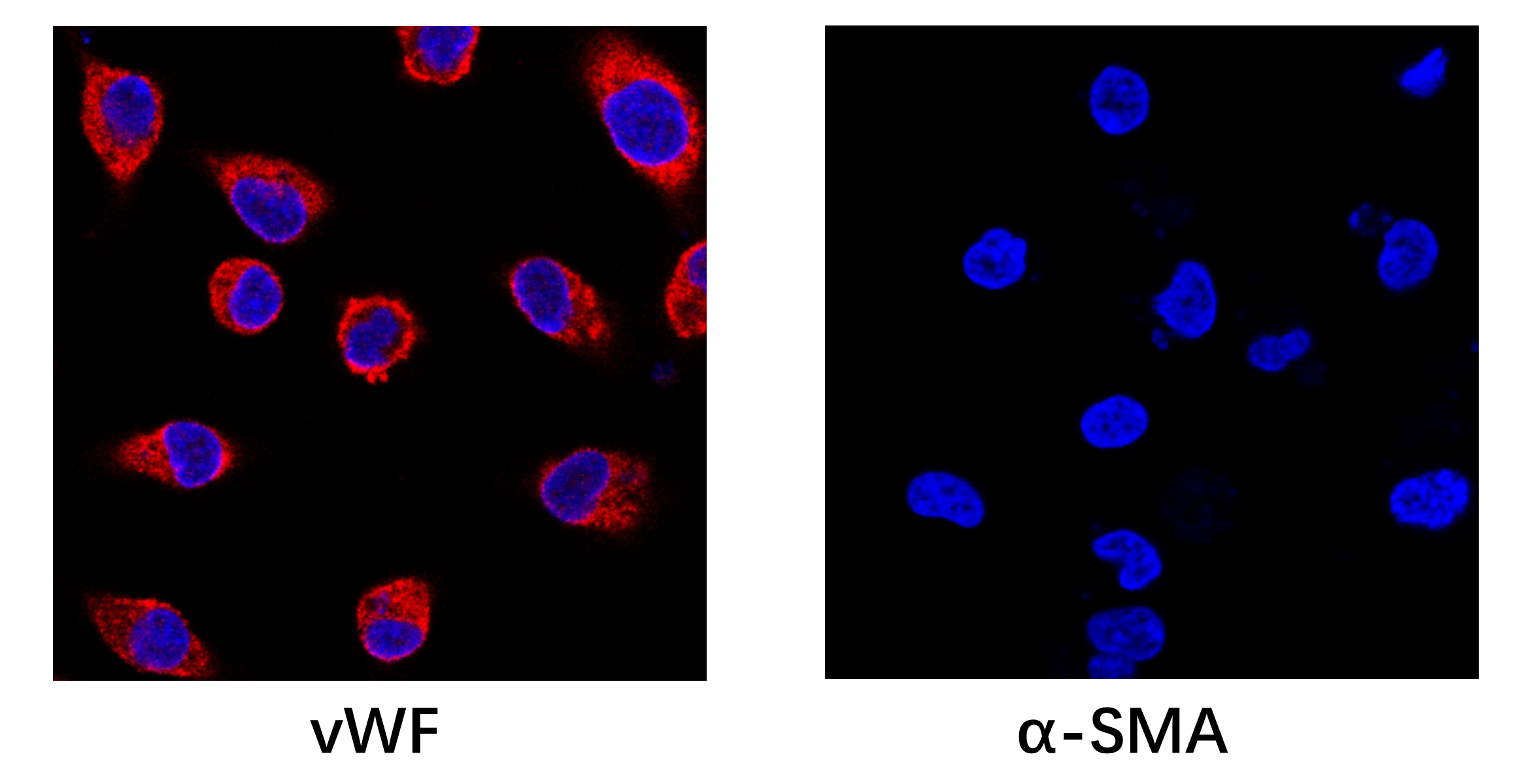

Supplement: Supplementary file 1 — Fig S1 [file JCMM-25-3601-s003.tif]

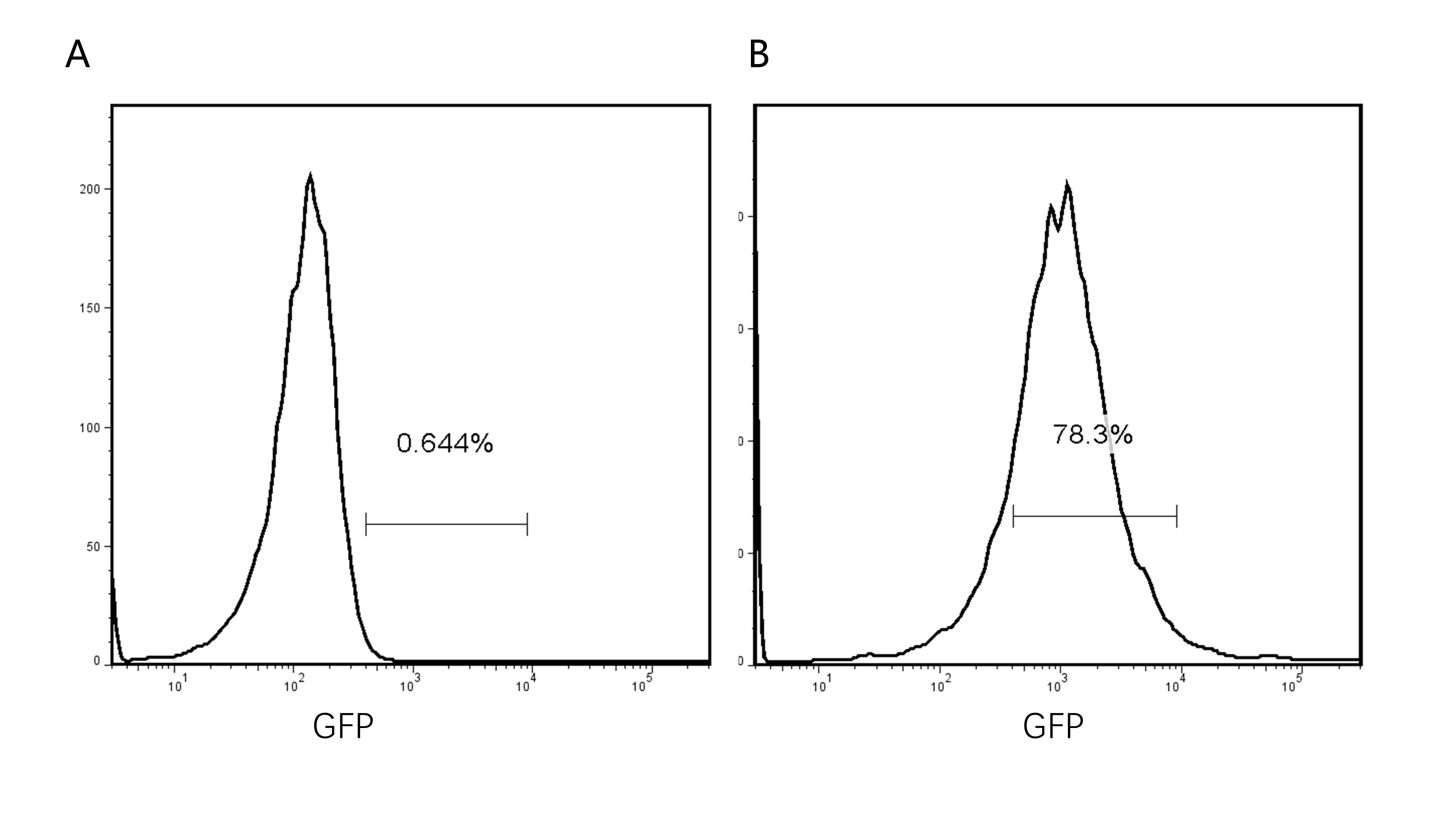

Supplement: Supplementary file 2 — Fig S2 [file JCMM-25-3601-s001.tif]

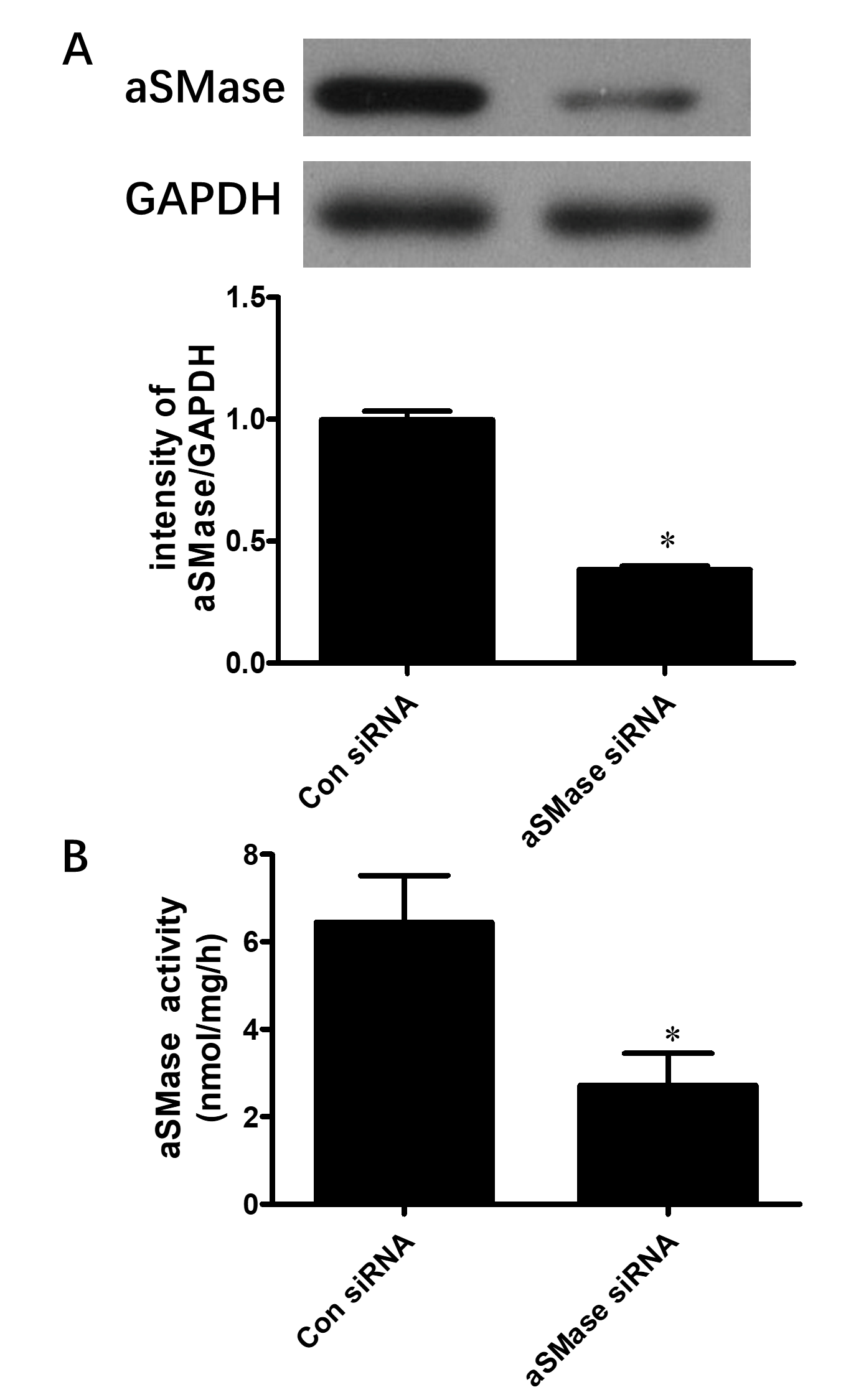

Supplement: Supplementary file 3 — Fig S3 [file JCMM-25-3601-s002.tif]

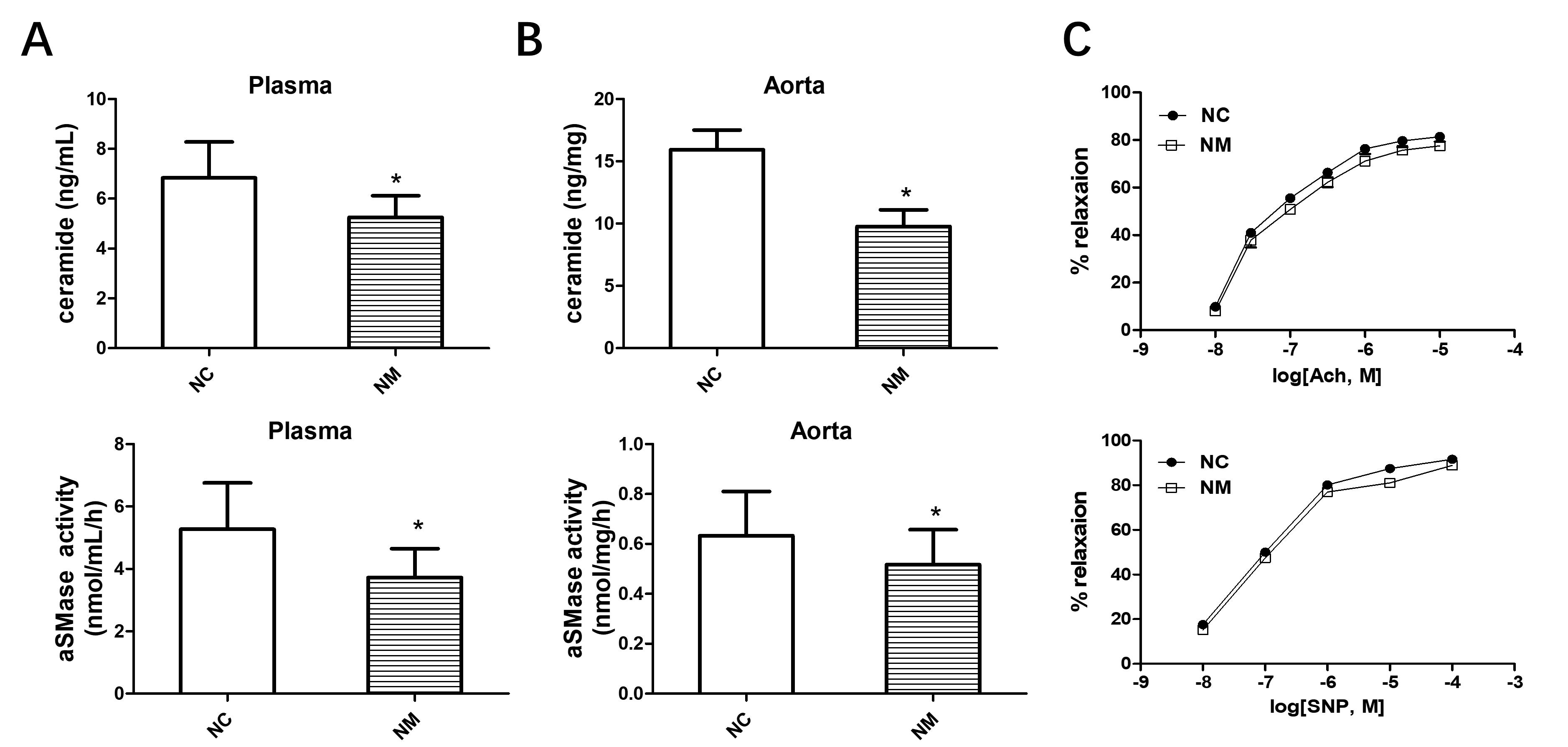

Supplement: Supplementary file 4 — Fig S4 [file JCMM-25-3601-s005.tif]

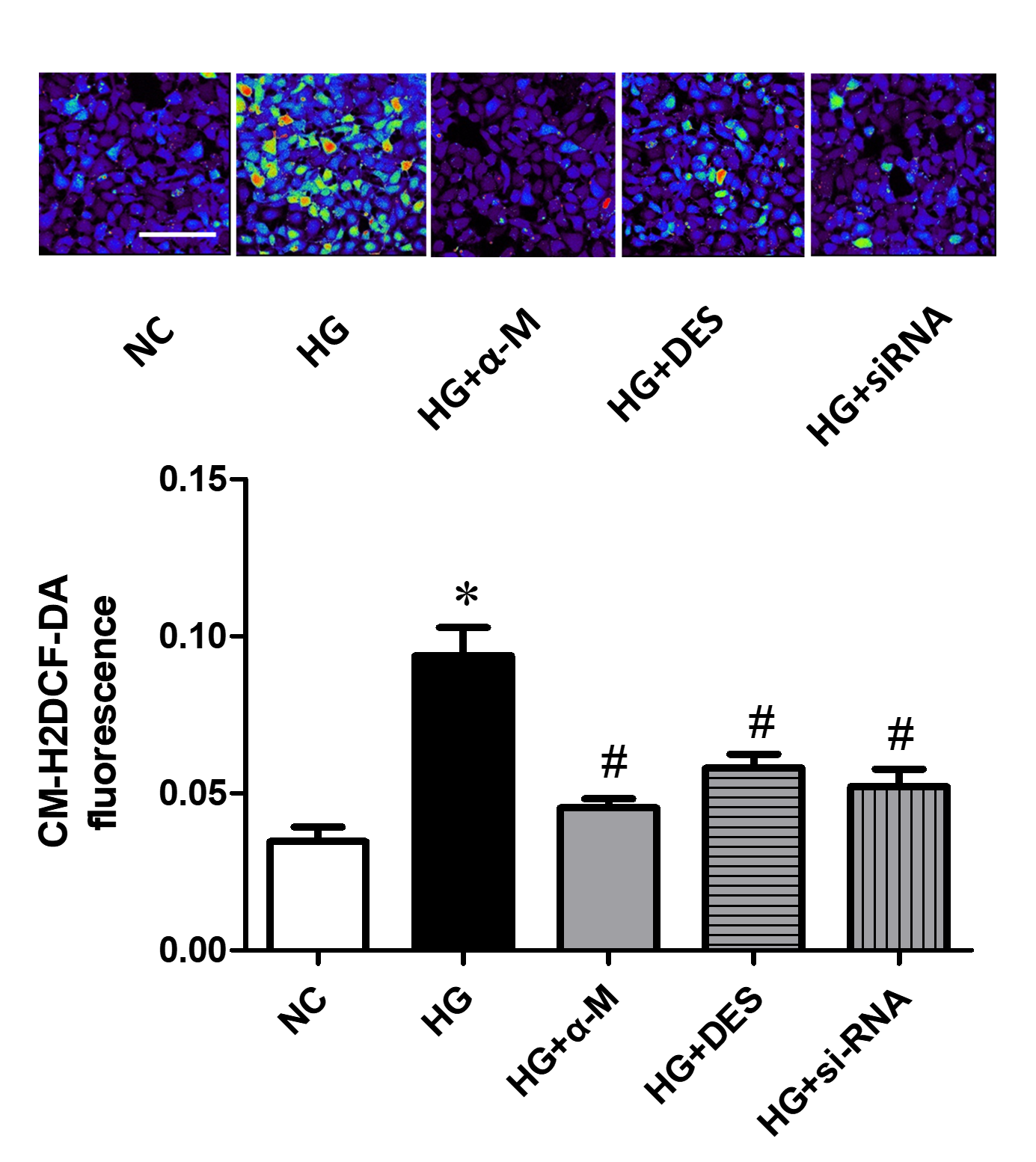

Supplement: Supplementary file 5 — Fig S5 [file JCMM-25-3601-s004.tif]
